# Identification ACTA2 and KDR as key proteins for prognosis of PD‐1/PD‐L1 blockade therapy in melanoma

**DOI:** 10.1002/ame2.12154

**Published:** 2021-03-23

**Authors:** Yuchen Wang, Zhaojun Li, Zhihui Zhang, Xiaoguang Chen

**Affiliations:** ^1^ State Key Laboratory of Bioactive Substances and Functions of Natural Medicines Institute of Materia Medica Chinese Academy of Medical Sciences and Peking Union Medical College Beijing China; ^2^ Beijing Key Laboratory of New Drug Mechanisms and Pharmacological Evaluation Study Institute of Materia Medica Chinese Academy of Medical Sciences and Peking Union Medical College Beijing China

**Keywords:** expression profiling data, hub genes, melanoma, PD‐1/PD‐L1 blockade therapy

## Abstract

Programmed cell death protein 1 (PD‐1) /programmed cell death ligand 1 (PD‐L1) blockade is an important therapeutic strategy for melanoma, despite its low clinical response. It is important to identify genes and pathways that may reflect the clinical outcomes of this therapy in patients. We analyzed clinical dataset GSE96619, which contains clinical information from five melanoma patients before and after anti‐PD‐1 therapy (five pairs of data). We identified 704 DEGs using these five pairs of data, and then the number of DEGs was narrowed down to 286 in patients who responded to treatment. Next, we performed KEGG pathway enrichment and constructed a DEG‐associated protein‐protein interaction network. Smooth muscle actin 2 (ACTA2) and tyrosine kinase growth factor receptor (KDR) were identified as the hub genes, which were significantly downregulated in the tumor tissue of the two patients who responded to treatment. To confirm our analysis, we demonstrated similar expression tendency to the clinical data for the two hub genes in a B16F10 subcutaneous xenograft model. This study demonstrates that ACTA2 and KDR are valuable responsive markers for PD‐1/PD‐L1 blockade therapy.

## 1 INTRODUCTION

Tumors can change their microenvironment via expressing various functional proteins. This renders immune cells, such as antigen‐specific T cells, cannot recognize and eradicate tumor cells efficiently. Eventually, tumors escape attack by immune cells and continue aggressive growth through adaptive immune resistance. Thus, breaking adaptive immune resistance has become a general strategy for anti‐cancer therapy.^1^

Programmed cell death protein 1 (PD‐1) was first described as a member of the immunoglobulin gene superfamily.^2^ However, it was subsequently found that PD‐1 also interacts with one of its ligands, programmed cell death ligand 1 (PD‐L1), and negatively regulates T‐cell‐mediated immune response.^3^, ^4^, ^5^, ^6^, ^7^ Therefore, the PD‐1/PD‐L1 axis has become an important target for cancer immunotherapy. Since the first approval of pembrolizumab (Keytruda) for patients with advanced or unresectable melanoma was announced in September 2014^8^; the number of PD‐1/PD‐L1 inhibitors approved by the FDA has increased in recent years and PD‐1/PD‐L1 inhibitors, are being tested in clinical trials to extend their use to other solid tumors.^9^, ^10^, ^11^, ^12^, ^13^, ^14^

Although PD‐1/PD‐L1 blockade therapy has been a major advance in cancer immunotherapy, there are still some limitations, including the lack of a sensitive, accurate biomarker to predict response sensitivity and prognosis in cancer patients.^11^ For example, the objective response rates of atezolizumab are less than 30% in patients with advanced or metastatic urothelial carcinoma in whom the disease has progressed after platinum‐based chemotherapy.^15^ Thus, it is important to explore biomarkers that can be used to assess the clinical outcomes of PD‐1/PD‐L1 blockade therapy.

Gene chip and differential gene profiles are efficient techniques that can probe the gene expression patterns in a sample at a time point. Gene expression levels and profiles obtained from microarrays have been widely used for identifying differentially expressed genes (DEGs).^16^ The combination of gene expression microarrays and bioinformatics can be used to find potential biomarkers.^17^

In this study, based on published clinical datasets from five melanoma patients treated with anti‐PD‐1,^18^, ^19^ we screened for DEGs. Next, we analyzed the signaling pathways involved and constructed the protein‐protein interaction (PPI) network for the DEGs. The expression of the key genes identified was measured in relevant animal models to validate these analyses. Our findings provide potential biomarkers for supporting the clinical use of PD‐1/PD‐L1 blockade therapy.

## 2 MATERIALS AND METHODS

### 2.1 Gene expression profile data

The Gene Expression Omnibus (GEO) datasets at the National Center for Biotechnology Information are an international public repository that contains microarray, next‐generation sequencing and other formats of high‐throughput functional genomic data.^20^ The gene expression profile dataset, GSE96619, from GEO was used in the study. There were 10 biopsy specimens derived from five melanoma patients who responded (complete response/partial response, n = 2) or did not respond (progression, n = 3) to anti PD‐1 therapy. Tumor samples were obtained from patients receiving anti‐PD‐1 therapy. Samples were immediately fixed in formalin followed by paraffin embedding and processed for snap‐freezing in liquid nitrogen.^19^

### 2.2 Identification of DEGs

We used MetaboAnalyst (https://www.metaboanalyst.ca/) to identify DEGs. The fold change of the gene expression level was calculated. Only genes with a *P* <.05 and fold change >1.5 or <0.67 were regarded as DEGs.

### 2.3 Functional and pathway enrichment analysis of the DEGs

The significant GO terms and KEGG pathway enrichment analysis of the identified DEGs were performed by using DAVID with thresholds of significant functions and pathways of *P* <.05 and enrichment gene count of >5.^21^, ^22^ DAVID has been used for systematic and comprehensive analysis of massive lists of genes.

### 2.4 Establishment of PPI network, modular analysis, and pathway identification

The STRING v10 online tool was used to construct and visualize the PPI network.^23^ We mapped the DEGs into STRING, and interactions with the threshold of combined score of >0.4 were selected as significant. The network was captured and modified by Cytoscape (http://www.cytoscape.org/).^24^ MCODE in Cytoscape was used for integrating the complex PPI network into unified conceptual frameworks and calculating the node degree (numbers of interconnections to filter hub genes). The hub genes were then selected with a cutoff degree of ≥10.^25^

### 2.5 GEPIA analysis of gene expression

GEPIA (http://gepia.cancer‐pku.cn/) was used to validate the hub genes. GEPIA is an online tool based on the sequencing database for gene expression analysis between tumor and normal data from The Cancer Genome Atlas and the Genotype‐Tissue Expression programs. We used GEPIA for the preliminary exploration of the differences in tyrosine kinase growth factor receptor (KDR) and smooth muscle actin 2 (ACTA2) expression level and the survival rate between skin cutaneous melanoma (SKCM) and normal samples.^26^

### 2.6 Establishment of mouse xenograft model

Eight‐week‐old male C57BL/6 mice were purchased from Beijing Vital River Laboratory Animal Technology Co., Ltd. (Beijing Vital River Laboratory Animal Technology Co., Ltd., Beijing, China). On day 0, B16F10 mouse melanoma cells were harvested in saline, and 1.5 × 10^6^ cells in 0.2 mL saline were injected subcutaneously into the right flanks of each mouse. On day 1, treatment was initiated after the mice were assigned randomly to the control and experimental groups. In the control group, IgG (BE0093, Bio X Cell, West Lebanon, NH) was dissolved in saline for intraperitoneal treatment every 3 days. In the PD‐L1 mAb treatment group, 10 mg/kg anti‐mouse PD‐L1 (BE0101, Bio X Cell, West Lebanon, NH) antibody was dissolved in saline for intraperitoneal treatment every 3 days. For the CTX group, 60 mg/kg CTX was dissolved in saline for intraperitoneal treatment every 7 days. The Tumor volume was calculated as Tumor volume = π × *L* × *W* ^2^/6, in which *L* is the maximum length of the tumor and *W* is the maximum width of the tumor. Mice were sacrificed by CO2 asphyxiation. When the mice were sacrificed, the tumors were stripped and weighed. The tumor growth inhibition (TGI) was calculated as TGI = (1 − tumor weight_treatment_/tumor weight_vehicle_) × 100%. Statistical analysis was performed with GraphPad Prism 8.0 software and the significance level was evaluated with a one‐way ANOVA model. Studies involving mice were approved by the Experimental Animal Management and Welfare Committee at the Institute of Materia Medica, Peking Union Medical College.^27^, ^28^, ^29^, ^30^, ^31^

### 2.7 RT‐qPCR

Real‐time quantitative PCR (RT‐qPCR) with primers was conducted as previously described.^32^ Total RNA from mouse tumor tissues was extracted with TRIzol (Life Technologies Inc, Carlsbad, CA), according to the manufacturer's instructions. The RNA (10 μg) from each sample was then reversed‐transcribed to obtain the cDNA with a reverse transcript kit (ReverTra Ace qPCR RT Kit, Toyobo Inc, Tokyo, Japan). qRT‐PCR was performed using the THUNDERBIRD qPCR Mix (Toyobo Inc) with a sequence detector (ABI Prism 7900, Applied Biosystems, Foster City, CA, USA), the data were analyzed by the 2^−∆∆Ct^ method, and ∆Ct was adjusted by the house‐keeping gene β‐actin. The fold change in expression was calculated as 2∆Ct (Treated − Untreated). The primer sequences of ACTA2 were: forward 5′‐CCCAGACATCAGGGAGTAATGG‐3′, reverse 5′‐TCTATCGGATACTTCAGCGTCA‐3′. The primer sequences of KDR were: forward 5′‐TTTGGCAAATACAACCCTTCAGA‐3′ and reverse 5′‐GCTCCAGTATCATTTCCAACCA‐3′. The primer sequences of β‐actin were: forward 5′‐GTGACGTTGACATCCGTAAAGA‐3′ and reverse 5′‐GCCGGACTCATCGTACTCC‐3′. RT‐qPCR cycle conditions were as previously described.^33^, ^34^

### 2.8 IHC staining

Samples were processed for IHC by routine techniques, as previously described.^35^, ^36^ Xylene was used to dewax the paraffin‐embedded sections. The deparaffinized tissue sections were incubated with 3% H_2_O_2_ for 10 minutes at 37°C to quench the activity of endogenous peroxidase. Proteinase K was used to digest the sections for antigen retrieval. Slides were incubated overnight at 4°C with the primary antibody for ACTA2 (19245, Cell Signaling Technology, Danvers, MA); KDR (2479, Cell Signaling Technology); PD‐L1 (64988, Cell Signaling Technology); CD4 (25229, Cell Signaling Technology) and CD8α (98941, Cell Signaling Technology); secondary antibodies conjugated with horseradish peroxidase (ZSGB‐Bio, Inc, Beijing, China) were initially incubated for 40 minutes at room temperature.

### 2.9 Statistical analysis

Data were expressed as the mean ± SD of at least three independent experiments. Statistical analysis was carried out with Prism (version 7.0, Graph Pad, San Diego, CA). Statistical significance was inferred at *P* <.05.

## 3 RESULTS

### 3.1 Sample collection from the dataset

Clinical data from melanoma patients who received PD‐1/PD‐L1 blockade therapy was analyzed. The original microarray dataset GSE96619 from the GEO database contained five pairs of melanoma tissue samples (before and after receiving anti‐PD‐L1 therapy with atezolizumab) from five patients. Of these five patients, two responded (complete response/partial response, Pt1 and Pt2) to the treatment, and the other three did not respond (progression, Pt3, Pt4, and Pt5). Dataset GSE96619 was also analyzed in other publications with different aims.^18^, ^19^

### 3.2 Identification of DEGs and analysis of gene expression

**FIGURE 1:**
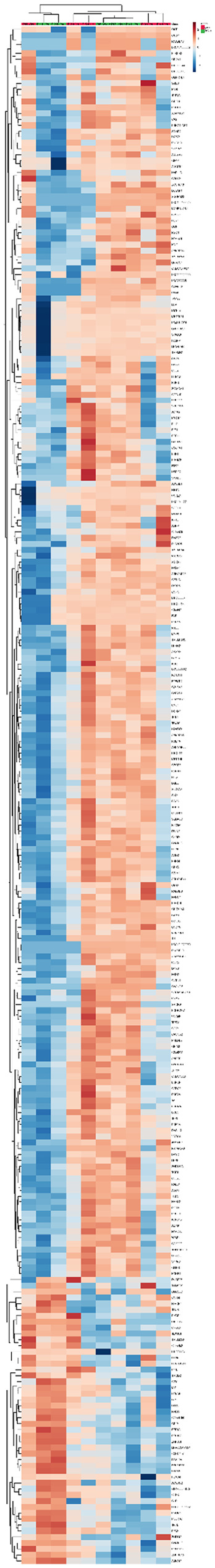
The heat map and cluster diagram of differential expression profiles of DEGs from dataset GSE96619 was developed. Each column represents an independent sample, and each row represents a gene. Blue represents the downregulated genes expression and red indicates the upregulated genes in each cell. The full figure with details is provided in Figure S1

We identified 704 DEGs involved in antitumor immunity by comparing expression in the responding group and no responding group using a *P*‐value of .05 (unpaired *t* test) as the criterion. Next, we set the criteria as *P* <.05 and fold change >1.5 or <.67, and 286 DEGs (232 upregulated and 54 downregulated genes) were identified in the responding group after the data from the treatment biopsy specimens were compared with their respective baselines (Table 1). MetaboAnalyst software was used to integrate the 286 DEGs in an expression heat map of the significant DEGs’ differential distribution (Figure 1). The full figure is provided in Figure S1.

**TABLE 1:** 286 differentially expressed genes (DEGs) were identified from GSE96619 dataset, including 232 up‐regulated genes and 54 down‐regulated genes in responding group on‐treatment (OnTx) biopsy specimens compared to their respective baselines (The up‐regulated genes were listed from the largest to the smallest of fold changes and down‐regulated genes were listed from the smallest to largest)

DEGs	Genes name
232 up‐regulated genes	CSNK1A1L, TH, LOC100505739, LOC101929124, FAM71A, RNASE10, GAS2L2, C9orf106, LOC101929696, PTX4, FLJ34503, CACNG5, LINC00696, LOC100133920, OR2A1, GDPD4, C6orf118, ELF5, PSPN, LOC101929369, SUPT20HL1, SATB2‐AS1, CDX2, BTBD18, ARL14, ZDHHC22, LOC101928227, PCDP1, SHISA3, SMCR9, TXNDC2, KRTAP5‐8, CGB7, GLIPR1L1, RNASE13, SNORA23, CBLN2, LINC00944, WDR49, TEX101, ANGPT4, LEP, C9orf47, HTR1B, RIMS1, METTL24, MMP27, BRSK2, NAP1L3, GRID1, GGN, KMO, SYBU, FKBP5, IMPG2, SPATA8, NKD2, GRIA3, LOC494127, NCAN, CD300E, ACTA2, DIRAS3, OMP, HLA‐DQB1, PCDHGB7, ADD2, VSTM4, SFTA1P, SNAP91, BNIPL, LINC00475, ANPEP, DUSP27, CCL2, ADAMTS3, BCL3, BMP6, TNFRSF10D, FGF11, CCL20, CECR6, SULT1C4, H2AFY2, GPX3, SIX2, SLC6A9, S1PR3, DLGAP1, RPLP0P2, PRKG1‐AS1, CDYL2, NDNF, PDE3A, OMG, LINC00115, LINC01106, LOXL1‐AS1, NHS, C15orf52, PROM2, TP53I11, ROR2, EFEMP2, CAMK1D, KCNJ15, THBD, ANKRD36, ARHGAP44, CA2, TFPI, CORT, ETS2, GFAP, RASL12, MALL, JAKMIP3, BCAM, ANKRD30A, FILIP1, NOXO1, ANO1, C16orf89, ENPEP, CCDC102B, CPT1A, COL4A1, NPDC1, FAM87B, LPHN2, UNC79, PDE2A, MARCO, LMF1‐AS1, METRNL, MANEA‐AS1, ARHGAP22, PCDH18, CHRD, DPYS, CCDC3, RUNX1, TBX15, WISP1, HS3ST1, COL4A2, FLT1, IFITM3, EDNRA, LOC653602, TCF4, FBLN5, CRYAB, FOXD4, SEMA3G, SOCS3, ADAMTSL2, ANTXR2, PLA2G5, IER5L, PODXL, CACNB2, MYCT1, RNF213, FAM26E, C2orf27A, C15orf59, TMEM253, ITGA1, PLXNA3, LEPR, CYP46A1, DPYD, NHSL2, TAL1, EBF1, ARHGAP23, UNC5A, PRR5L, SMAD6, MXD1, LINC00887, RASD2, C2CD2, TMEM150C, HIGD2B, HEY1, CNFN, PHLDB2, AQP9, TMEM154, UNC5B, PRR15, RAB31, RALGDS, LOC283335, SMAP2, GAMT, KCTD11, TMOD2, KDR, H2BFM, CRIM1, PROCR, EHBP1L1, KCNE4, COL8A2, MIDN, HSPG2, GLIPR1, DCHS1, EML1, GUCY1A3, ZC3HAV1, DSEL, PDE4A, CYP2S1, PIK3R1, KIF26A, ANKRD65, LRRCC1, TMEM52, ZCCHC18, ZNF385A, ZFP36, NBPF12, SGIP1, FN3K, TMEM249, ADAMTS2, AKAP2, PPIB
54 down‐regulated genes	GSTA7P, TMEM132D, PAH, TKTL1, ERICH6B, ZNF648, TMPRSS15, GPR119, NRTN, GS1‐259H13.2, PROM1, ACSBG2, CCDC13‐AS1, LOC101929690, CRYL1, TYMSOS, SERTAD4‐AS1, CTSK, RPL21P28, LYRM4, SGOL1, PPA2, MIF, RPS15A, EVPL, C20orf196, C1D, LRRC75A‐AS1, CNIH4, LOC100505592, MCM10, FLVCR1, CTSF, GPRC5D, CNTN1, GRIP1, LKAAEAR1, LINC01194, NFKBIL1, FAXC, CCDC167, RRM2, ADSSL1, TRUB1, KRT3, RPL23, RPL10A, ZDHHC15, POLR1C, EEF1A1, ASPM, ZNF581, CAMK1G, AGPAT1

### 3.3 Gene ontology analysis of DEGs

To understand the biological functions of our screened DEGs, we performed gene ontology (GO) analysis of the DEGs in DAVID (https://david.ncifcrf.gov/)^37^ with the criteria of *P* <.05 and count of ≥5. The DEGs were summarized into the following GO categories: “biological process” (BP; 145 GO terms), “molecular function” (MF; 13 GO terms), and “cellular component” (CC; 1 GO terms). Detailed information is provided in Table S1.

**FIGURE 2:**
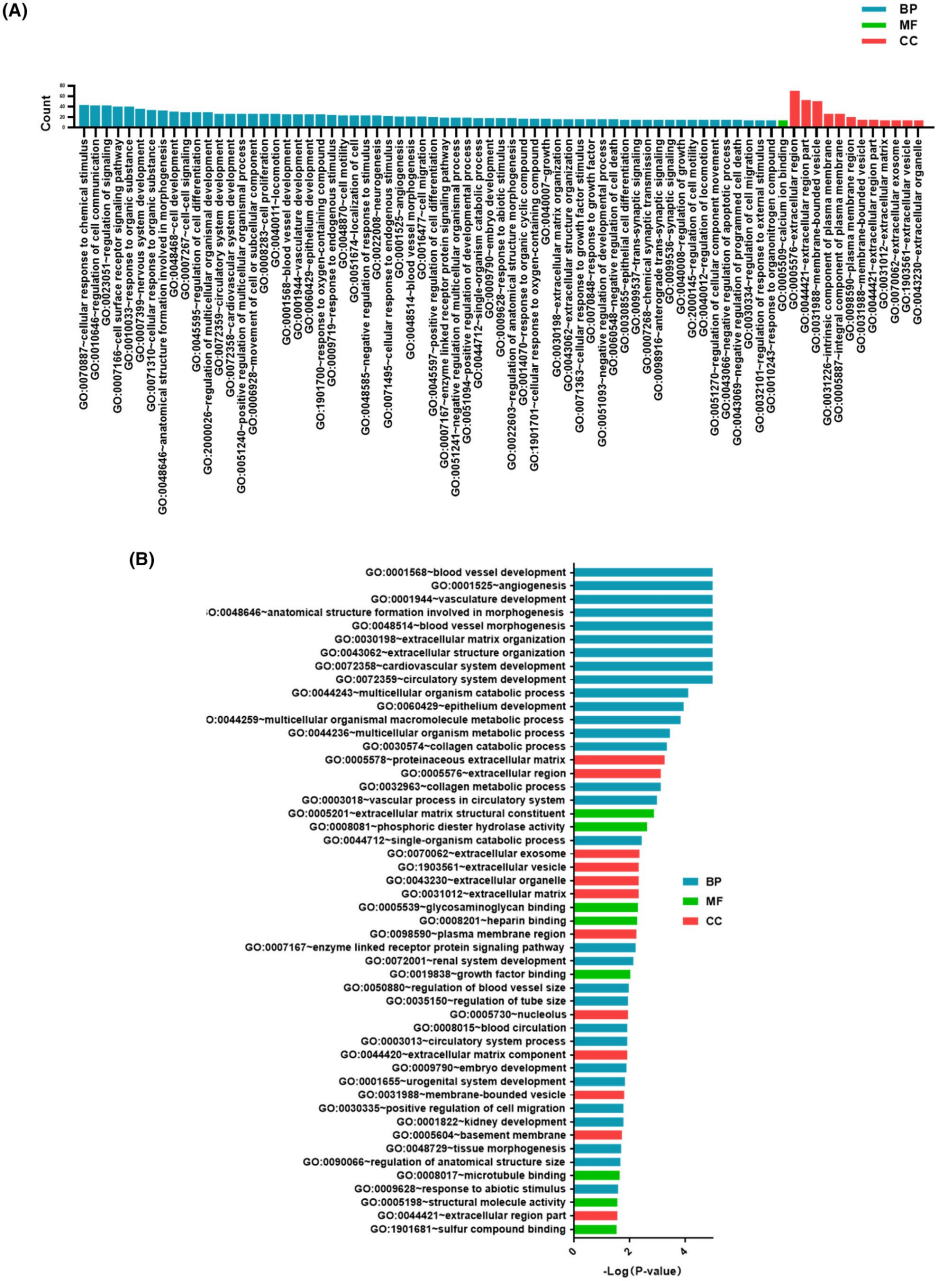
GO analysis and significantly enriched GO terms of DEGs. A, The 75 most significant GO terms for DEGs classified into three groups (ie, “molecular function”, “biological process”, and “cellular component”). B, The 50 most significantly enriched GO terms

In the BP group, the upregulated genes were mainly enriched in the “cellular response to chemical stimulus”, “regulation of cell communication”, and “regulation of signaling” GO terms. The downregulated genes were mainly concentrated in the “ncRNA metabolic process”, “carboxylic acid metabolic process”, and “oxoacid metabolic process” terms. In the MF group, the upregulated genes were mainly enriched in the “calcium ion binding”, “substrate‐specific channel activity”, and “channel activity” terms. The downregulated genes were mainly enriched in the “structural molecule activity” term. In the CC group, the upregulated genes were mainly enriched in the “extracellular region”, “extracellular region part”, and “membrane‐bounded vesicle” terms. The downregulated genes were mainly enriched in the “membrane‐bounded vesicle”, “extracellular region part”, and “extracellular exosomes” terms. The 75 most significant GO terms are shown in Figure 2A.

The “extracellular region” (70 involved genes), “extracellular region part” (53 involved genes), and “membrane‐bounded vesicle” (51 involved genes) GO terms in the CC group contained the majority of enriched DEGs. The 50 most significantly enriched GO terms are shown in Figure 2B, according to *P*‐value. The top five GO terms were “blood vessel development”, “angiogenesis”, “vasculature development”, “anatomical structure formation involved in morphogenesis”, and “blood vessel morphogenesis”. A total of 41 GO terms were located in the BP group, whereas only three GO terms were located in the MF group and six GO terms in the CC group.

### 3.4 KEGG pathway enrichment analysis of DEGs

We performed KEGG pathway enrichment analysis to elucidate the important pathways of DEGs. Enriched pathways of DEGs and detailed information are listed in Table 2. The KEGG pathway enrichment analysis of upregulated DEGs indicated that the important pathways were the “Ras signaling pathway” (eight genes), “TNF signaling pathway” (five genes), “adipocytokine signaling pathway” (four genes), and “prolactin signaling pathway” (four genes). The downregulated DEGs were mainly enriched in the “phenylalanine metabolism pathway” (two genes).

**TABLE 2:** Signaling pathway enrichment analysis of differentially expressed gene functions

Category	Term	Count	*P* value	Genes
Up‐regulated DEG				
KEGG_PATHWAY	hsa04014:Ras signaling pathway	8	.008201	FLT1, ETS2, FGF11, PLA2G5, PIK3R1, RALGDS, KDR, ANGPT4
KEGG_PATHWAY	hsa04668:TNF signaling pathway	5	.023185	CCL2, CCL20, SOCS3, BCL3, PIK3R1
KEGG_PATHWAY	hsa04920:Adipocytokine signaling pathway	4	.034874	LEP, SOCS3, LEPR, CPT1A
KEGG_PATHWAY	hsa04917:Prolactin signaling pathway	4	.036151	SOCS3, ELF5, TH, PIK3R1
Down‐regulated DEGs				
KEGG_PATHWAY	hsa00360:Phenylalanine metabolism	2	.048136	PAH, MIF

### 3.5 PPI network construction and hub gene identification

**FIGURE 3:**
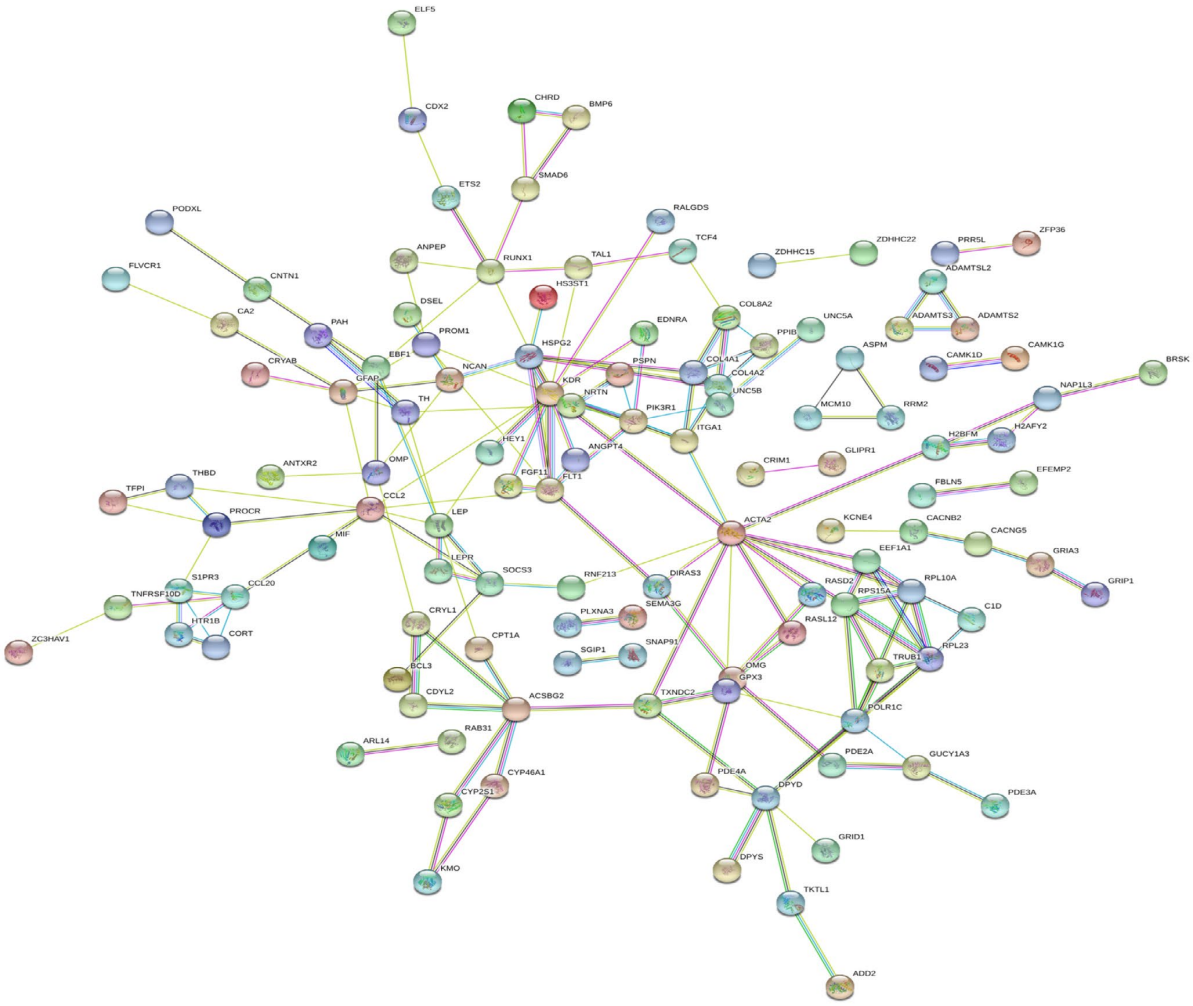
PPI network constructed with the DEGs. PPI enrichment *P* = .00383. Circles represent genes, lines represent the interaction of proteins between genes, and the results within the circle represent the structure of proteins. Line color represents evidence of the interaction between the proteins. Red line indicates the presence of fusion evidence. Green line ‐ neighborhood evidence. Blue line ‐ cooccurrence evidence. Purple line ‐ experimental evidence. Yellow line ‐ textmining evidence. Light blue line ‐ database evidence. Black line ‐ coexpression evidence

The data were imported into the STRING online database (http://string‐db.org), and proteins were linked by colored lines to indicate the different types of interaction evidence.^23^ The potential interactions of the DEGs were obtained by mapping the upregulated and downregulated DEGs in the STRING database. After removing the isolated nodes, a complex PPI network comprising 170 edges and 249 nodes was established (Figure 3). Each protein in the network is considered as node and the edges represent the predicted functional associations.^23^ The degree of a node represents the number of interactions between two nodes. Proteins closely associated with others in the network were identified with a degree of ≥10, and the hub genes, including, were identified KDR and ACTA2.

### 3.6 Modular analysis of the PPI network

**FIGURE 4:**
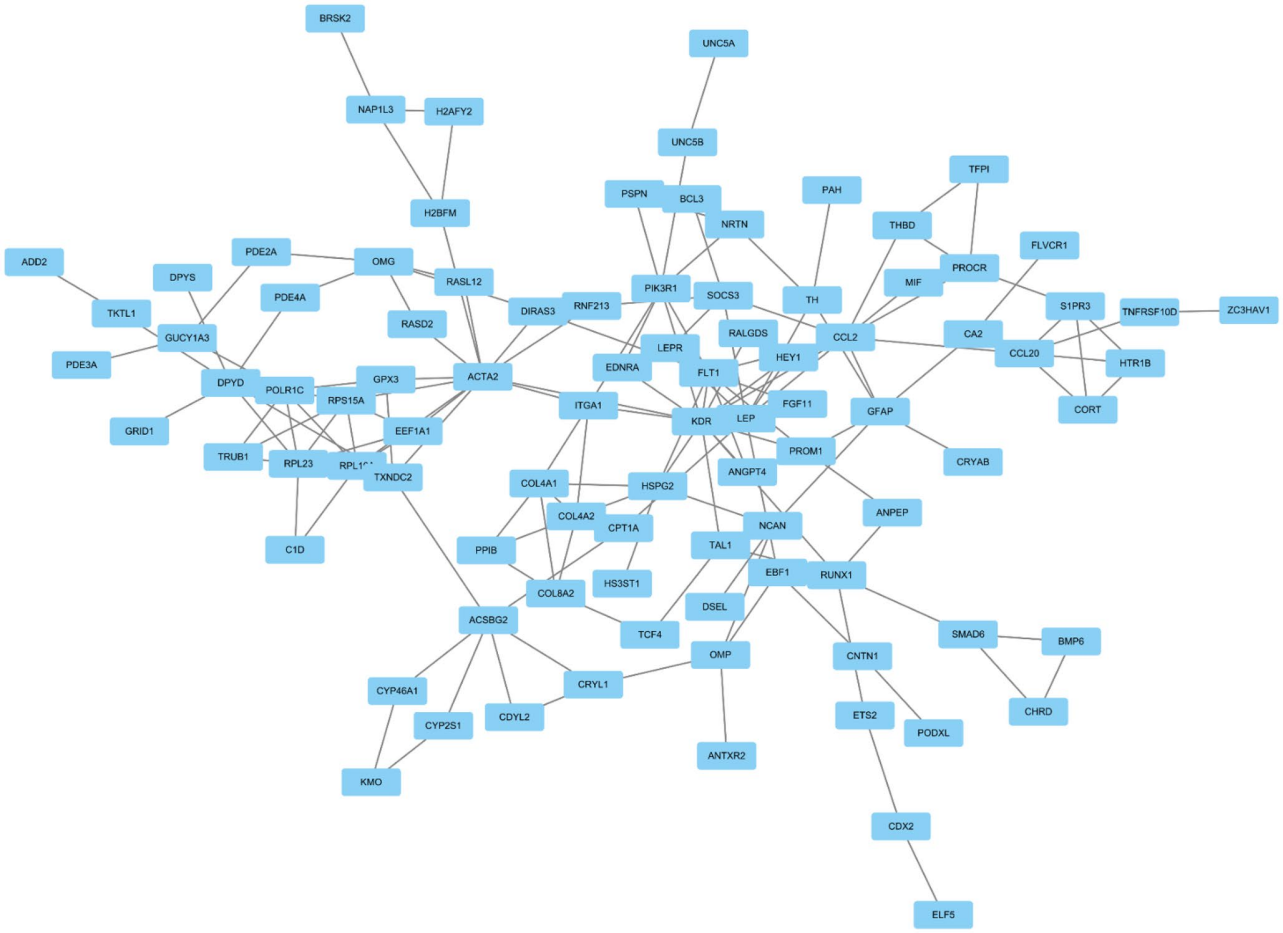
Modular analysis of the most significant module from the PPI network complex

Using Cytoscape Molecular Complex Detection (MCODE), the most significant module from the PPI network complex was selected, and the genes involved in the modules were analyzed (Figure 4).^25^ Enrichment analysis of the module showed that the genes were mainly associated with the “adipocytokine signaling pathway”, “TNF signaling pathway”, “Ras signaling pathway”, “prolactin signaling pathway”, “PI3K‐Akt signaling pathway”, “focal adhesion”, “Rap1 signaling pathway”, and “ECM‐receptor interaction”.

### 3.7 Verification and survival analysis of hub genes

**FIGURE 5:**
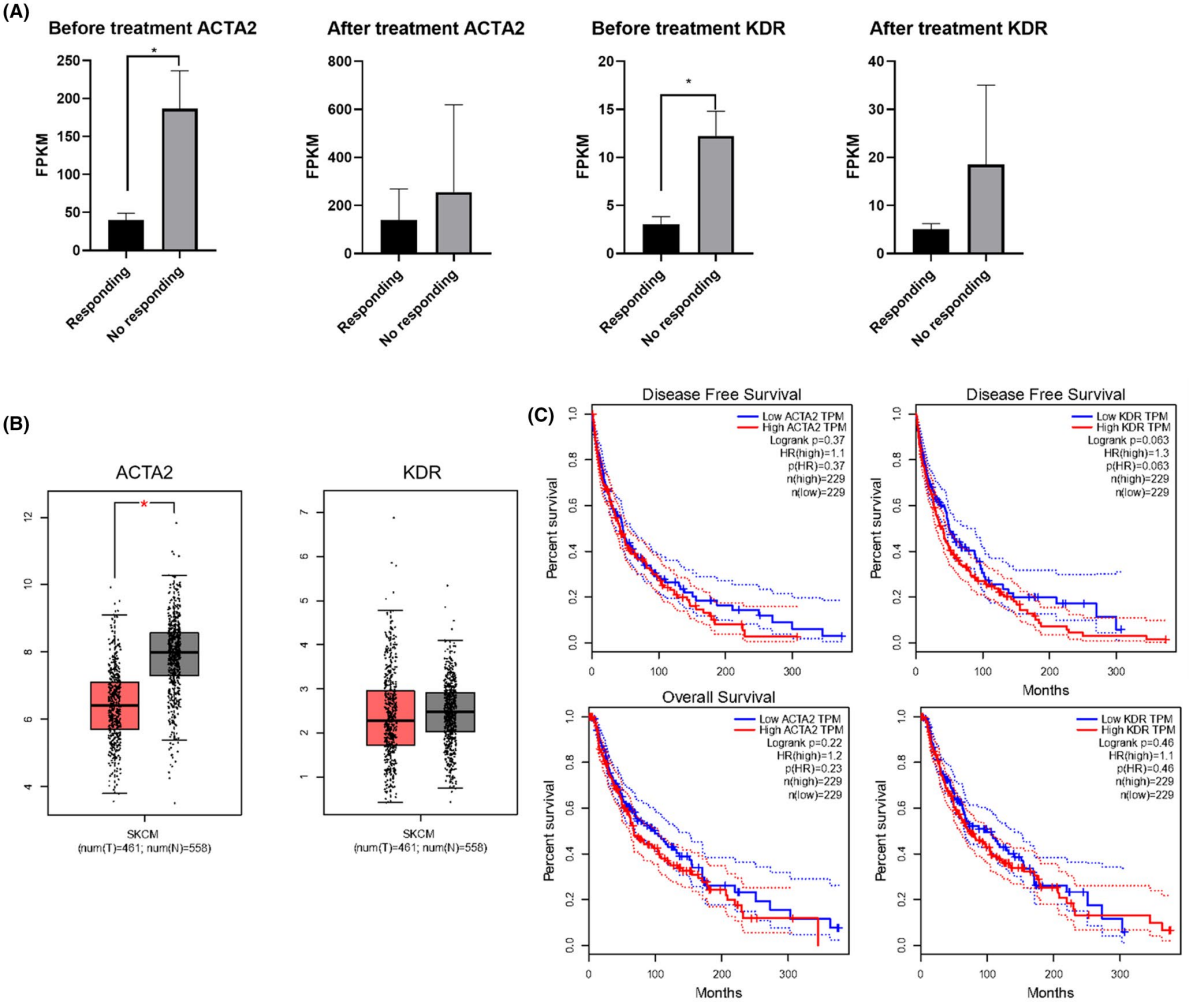
A, The expression analysis of KDR and ACTA2 in various conditionsfrom dataset GSE96619. B, Validation of hub genes in SKCM samples and normal tissue samples by the GAPIA database, red boxplots represent SKCM samples and Black boxplots represent normal tissue samples. C, Overall survival and disease‐free survival curves of hub genes KDR and ACTA2 in SKCM patients. Dashed line represents 95% confidence interval. (*P < .05)

The expression level of these two genes were analyzed from dataset GSE96619. In general, the expression levels of ACTA2 and KDR in no responding group was clearly higher than that in responding group regardless of either before or after the treatment (Figure 5A). Anti‐PD‐L1 treatment rendered the overexpression of ACTA2 and KDR in both no responding group and responding group. This indicates the overexpression of ACTA2 and KDR is a result of the Anti‐PD‐L1 treatment, but no significant expression difference is observed between no responding group and responding group. Next, Gene Expression Profiling Interactive Analysis (GEPIA) was used to analyze the contributions of the two hub genes in tumor tissues, overall survival and disease‐free survival period in SKCM. The expression level of the hub genes KDR and ACTA2 in tumor tissue were compared with their matched normal tissues, and survival curves were plotted (Figure 5). Comparing the expression of KDR and ACTA2 in the Oncomine database showed that the mRNA expression of ACTA2 was significantly different between SKCM and skin tissues (Figure 5B). To elucidate whether KDR and ACTA2 contributed to the survival period in patients with SKCM, we used GEPIA to analyze the overall survival and disease‐free survival for each hub gene. KDR and ACTA2 did not contribute to the overall survival and disease‐free survival (Figure 5C).

### 3.8 In vivo anti‐tumor study of PD‐L1 monoclonal antibody treatment

**FIGURE 6:**
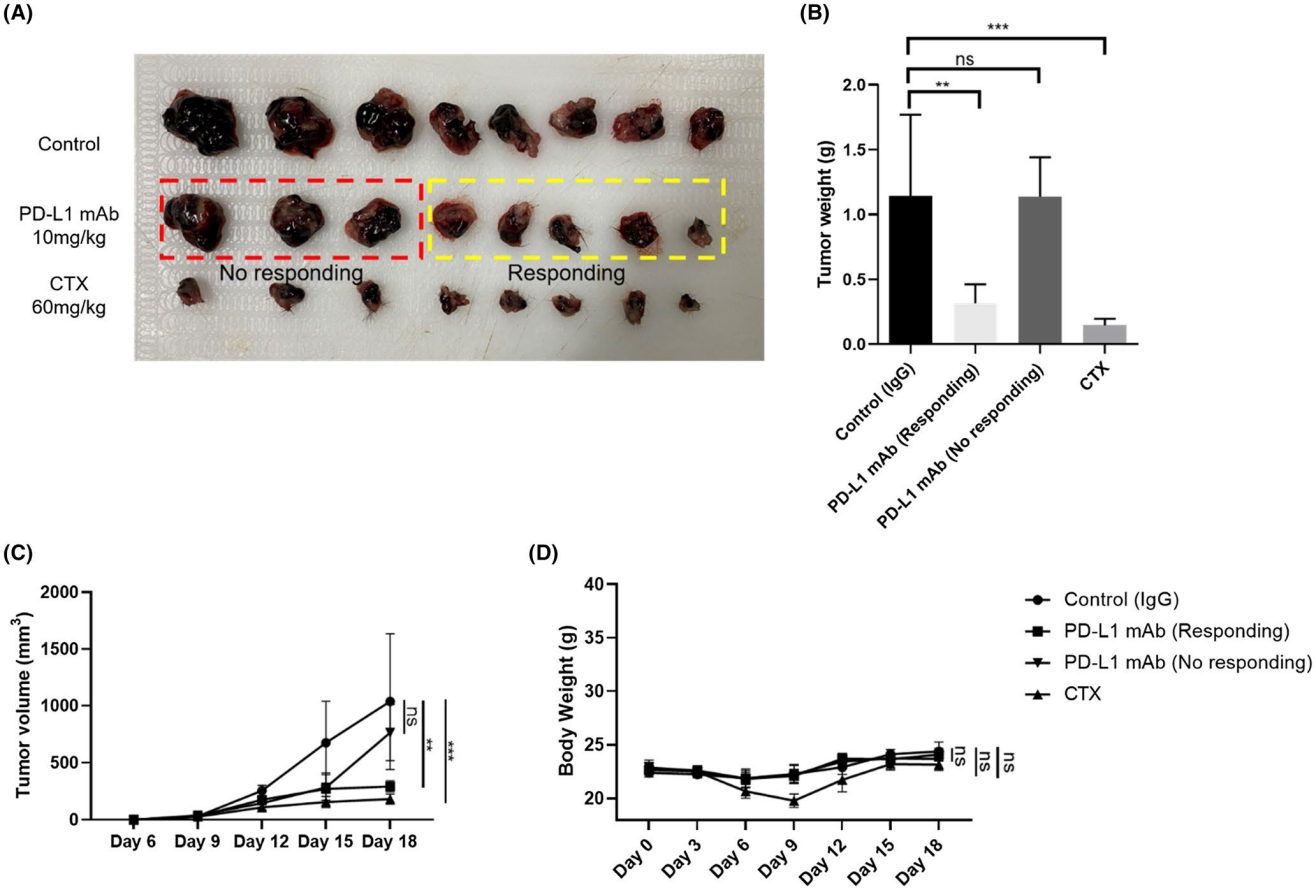
Anti‐tumor activity of PD‐L1 mAb treatment in the B16F10 xenograft mice model. A, Imaging of B16F10 tumor from xenograft mice. B, Tumor weights of the stripped tumors from xenograft mice (**P < .01, ***P < .001). C, Tumor volume changes following the treatment of IgG, PD‐L1 mAb and CTX. D, Average body weights of mice groups

The anti‐tumor effect of PD‐L1 monoclonal antibody (mAb) was evaluated in a B16F10 subcutaneous xenograft model in syngeneic mice. IgG was dissolved in saline to be used as the control. The anti‐cancer drug cyclophosphamide (CTX) was used as the reference. The average tumor weights was 0.625 g in the PD‐L1 mAb treatment group. We identified the individuals with lighter tumor weights than average tumor weight as responding group and the individuals with heavier tumor weights than average tumor weights as no responding group. Intraperitoneal administration (10 mg/kg/3 d) of PD‐L1 mAb significantly decreased the growth of melanoma B16F10 xenograft tumors in responding group. The responding and no responding group of PD‐L1 mAb treatment resulted in a 72.4% and 0.3% decrease in tumor weight compared with the control tumor after 18 days of treatment respectively (Figure 6, Table 3).

**TABLE 3:** B16F10 tumor inhibitory activity of PD‐L1 mAb treatment in vivo

Group	Dose	Number (End/Begin)	Body Weight (g) X ± SD	Tumor weight (g)	
				X ± SD	TGI
Control		8/8	24.38 ± 0.88	1.14 ± 0.63	NA
PD‐L1 mAb (responding)	10mg/kg	5/5	24.08 ± 0.58	0.32 ± 0.15	72.4**
PD‐L1 mAb (no responding)	10mg/kg	3/3	23.67 ± 0.12	1.14 ± 0.30	0.3
CTX	60mg/kg	8/8	23.15 ± 0.54	0.15 ± 0.05	87.3***

Abbreviations: NA, not applicable, TGI, tumor growth inhibition (100‐tumor weight of treatment group/control group × 100).

** *P* < .01.

*** *P* < .001.

### 3.9 Validation of the expression of hub genes in the B16F10 subcutaneous xenograft model

**FIGURE 7:**
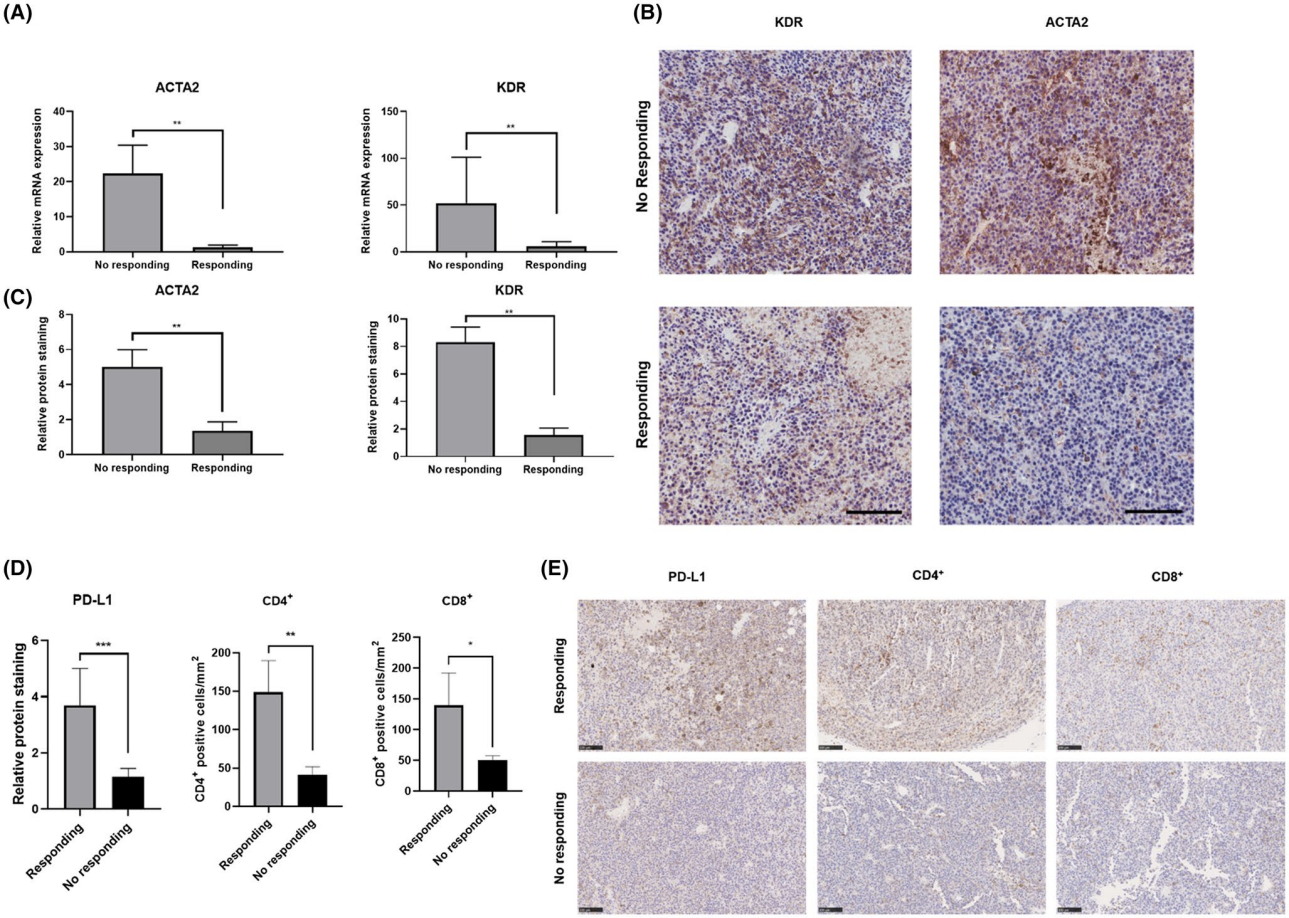
Validation of the expression of hub genes and PD‐L1; CD4^+^ and CD8^+^ cells in the B16F10 subcutaneous xenograft model. A, RT‐qPCR analysis of mRNA levels of ACTA2 and KDR in two groups of responding and no responding B16F10 subcutaneous xenograft model. B, Representative IHC image of protein expression of ACTA2 and KDR in two groups of responding and no responding B16F10 subcutaneous xenograft model (scale bar = 200 μm). C, Quantitation of IHC in tumor tissues showed as (B). D, Quantitation of IHC in tumor tissues showed as (E). E, Representative IHC image of protein expression of PD‐L1 and number of CD4^+^; CD8^+^ positive cells in two groups of responding and no responding B16F10 subcutaneous xenograft model (scale bar = 100 μm). Data are reported as mean ± SD from at least three independent experiments, which were performed in triplicate. (*P <.05, **P <.01, ***P <.001)

To examine the quality of our hub gene exploration, transcriptional and immunohistochemical (IHC) analyses of the expression of hub genes in the responding and no responding group of B16F10 subcutaneous xenograft model were compared (Figure 7). RT‐qPCR showed that the expression of the two hub genes was higher in the no responding group (Figure 7A). We verified the overexpression of the two hub genes by IHC staining. The expression of the two hub genes were significantly elevated in no responding individuals compared with responding group (Figure 7B,C). Expression level of PD‐L1 and CD4^+^; CD8^+^ positive cells were also detected by IHC staining. Both the expression level of PD‐L1 and number of CD4^+^ CD8^+^ positive cells were higher in the responding group (Figure 7D,E).

## 4 DISCUSSION

PD‐1/PD‐L1 blockade has achieved encouraging clinical outcomes in anti‐cancer therapy. However, expression level of PD‐L1 is not a necessary condition for the clinical application of PD‐1/PD‐L1 blockade drugs, and the six PD‐1/PD‐L1 blockade drugs approved by the FDA (nivolumab, pembrolizumab, atezolizumab, durvalumab, avelumab and Cemiplimab) have varied clinical performance. In patients with advanced melanoma with tumor PD‐L1 expression of less than 5% who received nivolumab, 114 of the 275 patients relapsed, whereas in patients with tumor PD‐L1 expression of more than 5%, only 31 of the 152 patients relapsed.^13^ The 12‐month recurrence‐free survival rate for pembrolizumab treatment in 853 resected stage III melanoma patients with PD‐L1 positive tumors was 77.1%, whereas in the placebo group, the 12‐month recurrence‐free survival rate was 62.6%. The effect of pembrolizumab was not related to the expression of PD‐L1.^10^ Atezolizumab is effective in patients with platinum‐treated locally advanced or metastatic urothelial carcinoma with all levels of PD‐L1 expression, and in patients with higher PD‐L1 expression on tumor‐infiltrating immune cells, atezolizumab showed a clear therapeutic benefit.^14^ Durvalumab is an effective adjuvant therapy for patients with stage III non‐small‐cell lung cancer after standard treatment.^9^ Patients with different PD‐L1 expression levels or Merkel cell polyomavirus statuses responded to avelumab.^12^ Lastly, the response to cemiplimab was observed no more than 50% in patients with advanced cutaneous squamous‐cell carcinoma.^38^ To understand treatment better, predictive biomarkers for evaluating the effectiveness of PD‐1/PD‐L1 blockade drugs should be identified.

Clinical research data is precious for providing a great deal of other important information that may have been irrelevant to the original analysis. Remining of these datasets of clinical research may help to identify the potential predictive biomarker.^39^ The GSE96619 dataset used in this study contains a balanced responding and no responding cases to anti PD‐1 therapy.^18^ Given that the statistical analysis is properly performed, the data can be used to obtain high‐quality results.

Our analysis identified the 50 most significantly enriched GO terms from dataset GSE96619, and the top five were “blood vessel development”, “angiogenesis”, “vasculature development”, “anatomical structure formation involved in morphogenesis”, and “blood vessel morphogenesis”. The terms are all related to biological functions of angiogenesis, which is crucial in tumors and is mainly induced by vascular endothelial growth factor (VEGF). VEGF plays a central role in promoting angiogenesis and suppressing tumor‐directed immune responses. Using an angiogenesis inhibitor to measure the suppressive state in the tumor microenvironment has become an attractive partnering strategy for immune checkpoint therapy.^40^

Through integrated bioinformatic analysis, we identified two hub genes, KDR and ACTA2. KDR, also called VEGFR receptor 2 (VEGFR2), encodes one of the two VEGF receptors (VEGFRs) and is a major regulator of vasculogenesis and angiogenesis.^41^, ^42^ VEGFs bind to VEGFRs, increasing capillary permeability and promoting vessel formation in endothelial cells.^43^ Moreover, there are possible functional correlations between PD‐1/PD‐L1 and VEGF/VEGFR. Atezolizumab (anti‐PD‐L1) in combination with bevacizumab (anti‐VEGF) is used as the first‐line treatment for advanced or metastatic hepatocellular carcinoma.^44^ In colon‐26 adenocarcinoma model, simultaneous blockade of PD‐1/PD‐L1 and VEGFR2 inhibited tumor growth synergistically.^45^ In addition, PD‐L1 expression is also significantly correlated with VEGF and microvessel density in patients with clear cell renal carcinoma^46^, ^47^ and classical Hodgkin lymphoma.^48^

ACTA2 maintains mechanical tension, cell shape, and movement, and thus may provide the dynamics of cytoskeletal structures for invasion and metastasis in tumors.^49^ In high‐risk breast cancer patients, relapse‐free survival was reduced when the expression levels of ACTA2, STAT1, and HER2 were increased. EGFR and HER2 dimerization modulated ACTA2 through the JAK2/STAT1 signaling pathway and ACTA2 gene abnormalities accelerated the invasion and metastasis of breast cancer cells.^50^ In addition, in patients with lung adenocarcinomas and high expression levels of ACTA2 in tumor cells, distant metastasis and unfavorable prognosis are increased substantially. Other in vitro experiments showed that migration, invasion, clonogenicity, and transendothelial penetration of lung adenocarcinoma cells were significantly impaired by downregulation of ACTA2. This indicated that ACTA2 may be a potential therapeutic target for metastatic lung adenocarcinoma.^49^

Our study in a mouse xenograft model also demonstrated that the ACTA2 and KDR hub genes were highly expressed in no responding group. In the mouse melanoma xenograft model, similar expression trendy of ACTA2 and KDR genes were observed to that in the clinical dataset. The correlation of the two hub genes with the prognosis predicted by our bioinformatic approach using the dataset is consistent with the biological validation data in a mouse xenograft model, which enhances the quality of our bioinformatic analysis. Thus, these genes can be used as the biomarkers for the accurate prediction of clinical outcomes of PD‐1/PD‐L1 blockade therapy. Notably, using GEPIA to analyze the contributions of the two hub genes to the overall survival and disease‐free survival period in SKCM showed that the two genes were not related to progression or survival of SKCM.

In conclusion, we performed a bioinformatics analysis using clinical dataset GSE96619. We screened 704 DEGs that may be relevant to PD‐1/PD‐L1 blockade therapy. By analyzing the GO and KEGG pathways, we found that DEGs were mainly enriched in “angiogenesis” terms, which provides a theoretical basis for PD‐1/PD‐L1 blockade therapy. We constructed a PPI network of DEGs and identified two hub genes (KDR and ACTA2) that could be potential biomarkers for predicting prognosis. We confirmed the identification by observing similar expression of the two hub genes in a B16F10 subcutaneous xenograft model and clinical samples. Our study revealed that ACTA2 and KDR could be used as responsive markers for PD‐1/PD‐L1 blockade therapy in melanoma.

## 5 ACKNOWLEDGEMENT

We appreciate the guidance on this project provided by Dr Yi Zhu and Dr Xu Zhang at Tianjin Medical University, Dr Qinghua Cui and Jiangcheng Shi at Peking University Health Science Center. We would like to thank Angel Garcia‐Diaz and colleagues for uploading and sharing their dataset GSE96619.

## CONFLICT OF INTEREST

The authors declare that they have no competing interests.

## AUTHORS’ CONTRIBUTIONS

YW carried out the study and drafted the manuscript, ZL collected and analysed data, ZZ did animal experiment, XC designed the project and revised the manuscript. All authors read and approved the final manuscript.

## ETHICS APPROVAL AND CONSENT TO PARTICIPATE

The study was reviewed and approved by the Experimental Animal Management and Welfare Committee at the Institute of Materia Medica, Peking Union Medical College.

## Supporting information

Fig S1

Table S1

## Data Availability

The datasets analysed during the current study are available from the GEO dataset GSE96619 repository, https://www.ncbi.nlm.nih.gov/geo/query/acc.cgi?acc=GSE96619.
