# Supplementary material for: Identification ACTA2 and KDR as key proteins for prognosis of PD‐1/PD‐L1 blockade therapy in melanoma
Source: Animal Model Exp Med. 2021 Mar 23;4(2):138–50. doi: 10.1002/ame2.12154 (PMC8212820; doi:10.1002/ame2.12154)
Supplement: Supplementary file 1 — Fig S1 [file AME2-4-138-s002.pdf]

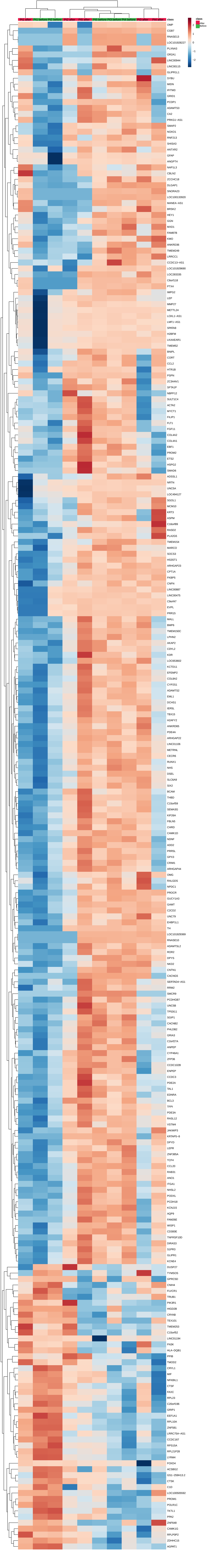

Figure S1. Detailed heat map and cluster diagram of differential expression profiles of DEGs from dataset GSE96619. All the 10 samples are hierarchically clustered into 2 groups, Pt1 OnTx, Pt1 baseline and Pt2 baseline clustered in one group and Pt2 OnTx, Pt5 OnTx, Pt5 baseline, Pt3 baseline, Pt4 baseline, Pt3 OnTx and Pt4 OnTx clustered in another group. Clustering pattern and color scale at the top indicates the magnitude of gene expression. In the heat map, each row reflects a single gene labeled with its name, each column reflects an independent sample from GSE96619. Genes from different samples are clustered together by their correlation coefficients. Expression levels higher than the mean are filled with red while those below the mean are filled with blue.
